# Associations between starting and stopping volunteering and physical activity among older adults - longitudinal evidence from the German Ageing Survey

**DOI:** 10.1186/s12889-022-12982-8

**Published:** 2022-03-23

**Authors:** Linda Baumbach, Hans-Helmut König, André Hajek

**Affiliations:** Department of Health Economics and Health Services Research, University Medical Center Hamburg-Eppendorf, Hamburg, Germany

**Keywords:** Physical activity, Volunteering, Voluntary work, Sex differences, Successful ageing

## Abstract

**Background:**

Physical activity (PA) contributes to healthy aging. Several studies have investigated factors influencing PA. However, population-based studies evaluating associations between volunteering and changes in PA are lacking. Our aim was to clarify whether starting and stopping to volunteer is associated with changes in physical activity in older adults.

**Method:**

We used data from the German Ageing Survey (wave 5 and 6 in the years 2014 and 2017), which is a representative survey of community-dwelling middle-aged and older adults. We included individuals ≥ 65 years (analytical sample: *n* = 5,682). PA was investigated using questions from the international physical activity questionnaire (IPAQ) and converted into metabolic equivalent of tasks (METs) per week. Changes in volunteering status in groups or organizations (yes/no) and their association with changes in PA were investigated in adjusted asymmetric fixed effects models stratified by sex.

**Results:**

We found an association, between starting to volunteer and increased physical activity in older adults in the total sample (ß = 1,078.93, *p* = 0.052). This change reached significance for men (ß = 1,751.54, *p* = 0.016), but not for women (ß = 187.25, *p* = 0.832) in the stratified analyses. In the total sample, there was no association between stopping volunteering and decreases in PA (ß = -285.61, *p* = 0.543). This also held true in the stratified analyses for men (ß = -320.76, *p* = 0.583) and women (ß = -158.96, *p* = 0.845).

**Conclusion:**

Our study identified an association between beginning to volunteer and increased physical activity among older men. Thus, beginning to volunteer may assist older men in increasing their physical activity levels.

## Key Points

- First observational study: Examining the link between changes in volunteering status and physical activity in late life
- Longitudinal data from a nationally representative sample were used
- Older men, but not older women, who started to volunteer, increased their physical activity
- Stopping volunteering was not associated with changes in physical activity
- Efforts to start volunteering among older men may assist in increasing their physical activity levels in the long-term

## Introduction

With an increasing proportion of society comprising older adults, active, health living and ageing is desirable. Physical activity (PA) supports healthy aging, as it prevents, and is recommended as treatment for, several chronic conditions [1, 2]. Even if PA is taken up relatively late in life, it still positively affects health [3]. Nonetheless, the majority of older adults do not meet the physical activity levels recommended by the WHO (World Health Organisation) [4].

An increase in PA in older adults is difficult to achieve [5]. Interventions that include social support have been evaluated to their impact on increasing PA [6]. Individual related determinants of PA have been investigated in recent decades and several related literature reviews have been published [7–9]. They have found, among other things, that an adult’s age and health status were associated with participation in physical activities. However, whether volunteering is associated with physical activity levels has not yet been conclusively evaluated [10].

According to an announcement of the German Ministry for Family affairs, Senior citizens, Women and Youth (BMFSFJ) in the beginning of 2021 [11], around 40% of people above 14 years in Germany volunteer. In adults aged 65 years or older, the proportion was lower (31%) [12]. 2019 was the first year where there was no statistically significant difference in undertaking volunteering between men and women [11]. However, it was not reported whether this holds true in older adults. The time spent volunteering varied significantly. 60% of volunteers spend up to two hours per week volunteering, and 17% volunteered for six or more hours per week [11]. Volunteering is, among other things, associated with increased self-rated health and life satisfaction in older adults [9, 13].

Whether volunteering, and in particular starting and stopping to volunteer, is associated with changes in physical activity in the population has not been conclusively studied, neither for men nor for women [10]. For example, in an RCT project, a positive association between volunteering at a school for at least 15 h per week and PA was found [14]. However, it is unclear whether the findings can be generalized to any type of volunteering, and if both starting and stopping to volunteer is associated with changes in physical activity. We therefore aimed to investigate associations between starting and stopping volunteering and changes in PA in a representative sample of older adults, with a focus on differences between sex.

We assume that starting to volunteer is positively associated with changes in PA, as volunteering can result in social support, which is associated with increased participation in PA [15]. A difference between women and men might be present due to the differences in performed tasks. In an Australian study, it was found that men are more likely to engage in volunteering with sports and recreational organisations, which typically involve more physical activity, as opposed to volunteering in religious organisations, which is preferred by women [16].

Knowledge about the associations between changes in volunteering status and changes in PA would be relevant, among other things, for general practitioners, physiotherapists, geriatrists, and volunteering organisations. Health professionals may use such sex specific information to advise older adults on how to support their healthy living and aging. Volunteering organisations may use findings to motivate and recruit new volunteers.

## Methods

### Data source (study design – setting –)

We used data from the nationwide representative German Ageing Survey (DEAS – “Deutscher Alterssurvey”). The survey is funded by the BMFSFJ and has been organized by the German Centre of Gerontology (DZA – “Deutsches Zentrum für Altersfragen”) since 2002. Participants who were 40 years or older were recruited for the first time in 1996 from population registers within municipalities. If participants provided written consent, they were asked to participate again in 2002, 2008, 2011, 2014, and 2017. In 2002, 2008, and 2014 new randomly selected samples were added to the DEAS. Consequently, the survey is a cross-sectional and longitudinal study of the community-dwelling population. The aim of the DEAS is, on the one hand, to allow for the evaluation of living conditions, and on the other hand, to evaluate the consequences of aging on individuals living in Germany. The survey includes a Computer-Assisted Personal Interview (CAPI) conducted by trained interviewers at the participant’s home, as well as from a questionnaire, which was given to the participant after the interview. The survey obtains data related socio-economic-demographic circumstances, living conditions, and other topics related to aging via the CAPI. Data related to subjective attitudes, psychological topics, and health information is obtained via the questionnaire filled out by the participant. The questions asked are adapted on an ongoing basis to respond to research needs and emerging evidence.

We used data from 2014 and 2017, as our outcome variable of interest was first added in 2014. The sample in 2014 and 2017 covered data from 10,324 and 6,626 participants respectively. In accordance with the aim of this study, we included participants who were at least 65 years when they participated in 2014. Amongst all participants the response rate of the DEAS is about 30% [17], which is in line with the response rate of other German surveys. In comparison to other European studies on aging, however, it is lower [17, 18]. The most common reasons for missing follow up data were time and health limitations, as well as refusal to participate [19]. Additional, detailed information on the DEAS survey has been reported elsewhere [17]. The study follows the recommendations of the Helsinki Declaration. All respondents provided informed consent and were offered small incentives to participate.

### Variables

#### Dependent variable

Our dependent variable of interest was change in PA from 2014 to 2017. In line with various previous studies, we used the “International Physical Activity Survey” (IPAQ) to quantify physical activity [20, 21]. The IPAQ measures physical activity in metabolic equivalent of task (MET) [22–24]. Participants answered three multiple choice questions: *“How often do you practice 1) vigorous, 2) moderate, 3) low physical activities?”*. Appended to each question, related changes in breathing *(1) much harder than normal, 2) a bit harder than normal, 3) normal* and examples of PA types e.g. *1) fast cycling, 2) hiking, 3) a walk as relaxation*, were given. Participants were reminded to consider activities they perform as part of their daily living, such as gardening, for vigorous and moderate activities. In the first response step, participants could choose between six answer options: *daily, several times per week, once a week, 1–3 times per month, less frequent,* or *never*. If participants answered at *least once a week* they were asked in a second response step how many hours and minutes they performed this intensity of PA per week. If patients answered less than *once a week,* we coded this PA intensity with no minutes per week. We coded the responses on the three intensities of PA in accordance with the IPAQ to MET per week [24]. Consequently, participants whose average minutes of combined physical activity intensities per day exceeded 960 min (16 h) were excluded. One minute of vigorous, moderate, and low physical activity accounted for 8, 4, and 3.3 METs respectively. Thus, as an example, to reach 1000 MET a participant could spend 4 h and 10 min in moderate physical activities or about 5 h in light physical activities, or any appropriate combination.

#### Independent variables (exposures)

Our independent variable of interest was the change in volunteering status *(starting/stopping volunteering)*. Participants were asked if they perform an honorary duty in a group or organization in which they are a member. As in previous studies the response was coded as *yes, volunteering* vs. *not volunteering* [10, 25].

#### Potential confounder variables

Based on a literature search, we identified and included eight potential confounding variables in our regression models [7, 9, 10, 13, 26].

We accounted for socioeconomic factors including age, marital status, and monthly equivalence income. General self-efficacy was included as a psychological factor. Self-rated health, sum of morbidities, physical functioning and experience of an accident or illness in the last 3 years were included as health-related factors in our model.

Marital status comprised five categories*: being married and living together, being married and living apart, divorced, widowed,* or *single*. The monthly equivalence income was measured using the Organisation for Economic Cooperation and Development (OECD) equivalence scale. Generalized self-efficacy was measured using the scale from Schwarzer und Jerusalem (1995) ranging from 1 (low) to 4 (high). The original tool consists of ten statements such as *“I can always manage to solve difficult problems if I try hard enough”*. Participants respond to each statement on a four-point scale ranging from 1 = not at all true to 4 = exactly true. For the DEAS study, a short version was used. It consists of only five statements and was constructed in co-operation with Ralf Schwarzer. Self-rated health was measured using a five-point scale ranging from 1 = very good to 5 = very bad. The sum of morbidities considered the following eleven self-reported diseases *(yes/no)*, which we assumed to potentially influence physical activity and volunteering: 1) Cardiac and circulatory disorders, 2) Bad circulation, 3) Joint, bone, spinal or back problems, 4) Respiratory problems, asthma, shortness of breath, 5) Stomach and intestinal problems, 6) Cancer, 7) Diabetes, 8) Gall bladder, liver or kidney problems, 9) Bladder problems, 10) Eye problems, vision impairment, and 11) Ear problems, hearing problems.

Physical functioning was quantified using the short form of the SF-36 health survey [27]. Participants quantify their difficulties in performing ten tasks from 1 = major limitation to 3 = no limitations at all. The results are converted to a score from 0 = low to 100 = high physical functioning. Finally, respondents’ reported incidence of an accident or major disease in the last 3 years using one of the following items: *yes—an accident, yes—a disease, yes – both,* or *no*.

### Statistical methods

We used asymmetric fixed effects (FE) regressions to estimate the association between change in volunteering status and change in physical activity levels. These models investigate associations between a change in one variable and a change in another variable within individuals. Within our study, we examined the association between having commenced volunteering (i.e., the FE model included the change from not volunteering to volunteering within individuals over time) and changes in physical activity within individuals over time. An advantage of FE regressions over other widely used regression models is a reduction in the risk of bias due to unobserved variables, since all time-constant variables do not bias the estimates [28]. In models like random effects regression, which performs evaluations across individuals, unobserved time-constant variables, such as genetic disposition, which are correlated with the independent variable of interest, lead to inconsistent estimates [28]. Estimates of the FE regression, however, are consistent when such correlations are present [28]. The estimates from FE regressions are based on within-individual changes, thus time constant variables, such as sex and genetic factors, do not influence the results. As a consequence, in FE regressions it is not possible to estimate the association between time-constant variables and their outcome, however they may be used for stratification, and can be included as moderating factors (e.g. sex x physical activity) in these models [28]. Moreover, asymmetric FE regressions, can differentiate between the direction of a change in the independent variable e.g., starting vs. stopping volunteering [29].

We performed unadjusted and adjusted asymmetric FE regressions stratified by sex. All analyses were performed in Stata 16.1 (StataCorp., College Station, Texas, USA).

## Results

### Participants

In our analytical sample, 5,682 participants were included (3,172 were men and 2,510 were women).

The majority of the participants were married and living together with their partner. Further characteristics of the participants stratified by sex are given in Table 1.

**Table 1:** Characteristics of observations included in the fixed effects models

	**Men (*n* = 3,172)**	**Women (*n* = 2,510)**
**Age, mean (SD)**	74.1 (6.0)	73.2 (5.9)
**Marital status,** *n* **(%)**		
- Married living together	2511 (79.2%)	1431 (57.0%)
- Married living apart	43 (1.4%)	19 (0.8%)
- Divorced	186 (5.9%)	260 (10.4%)
- Widowed	332 (10.5%)	700 (27.9%)
- Single	100 (3.2%)	100 (4.0%)
**OECD monthly equivalence income, mean (SD)**	1951.2 (1127.5)	1835.5 (1347.7)
**Self-efficacy, mean (SD)** from 1 (low) to 4 (high)	3.1 (0.4)	3.1 (0.4)
**Self-rated health,** *n* **(%)**		
- Very good	192 (6.1%)	180 (7.2%)
- Good	1416 (44.6%)	1112 (44.3%)
- Average	1235 (38.9%)	971 (38.7)
- Bad	278 (8.8%)	210 (8.4%)
- Very bad	51 (1.6%)	27 (1.5%)
**Sum of morbidities, mean (SD)** from 0 to 11	3.1 (2.0)	3.0 (2.0)
**Physical functioning (SF-36 subscale), mean (SD)** from 0 (worst) to 100 (best)	80.3 (22.9)	75.0 (25.6)
**Incidence of accident or disease, n (%)**		
- Disease	756 (23.8%)	461 (18.4%)
- Accident	157 (4.9%)	228 (9.1%)
- Both	46 (1.5%)	35 (1.4%)
- No	2213 (69.8%)	1786 (71.2%)
**Physical activity measured in MET per week**	5759.6 (5259.3)	6017.1 (5579.2)
**Volunteering in an organisation,** ***n *(%)**	770 (24.3%)	499 (19.9%)

*MET* Metabolic Equivalent of Task, *OECD* Organisation for Economic Cooperation and Development, *SD* standard deviation

Missing values on the characteristics of interest were evaluated for the included participants in 2017 For all variables, missing values were markedly lower than 1% (e.g. general self-efficacy 0.3%). However, for the variables physical activity intensity (2%) and income (4%), the proportion of missing values were slightly higher.

Changes in volunteering status were observed for 236 men and 177 women. In sum, 151 men stopped and 85 started volunteering. 108 and 69 women stopped and started volunteering respectively.

### Regression analysis

When comparing the effect size of changes in physical activity between starting and stopping volunteering, there was a statistically significant difference observed within men (*p* = 0.041) but not in women (*p* = 0.953).

In the unadjusted asymmetric FE regressions including all participants, starting (ß = 1048.87, *p* = 0.041) but not stopping (ß = -649.17, *p* = 0.104) volunteering was associated with changes in PA in older adults. Thus, roughly translating the beta coefficient of METs into PA per week, participants who started to volunteer increase their PA by eg. about 4.5 h of moderate PA. In the unadjusted analyses stratified by sex, the statistically significant association between starting to volunteer and PA remained significant in men (ß = 1689.26, *p* = 0.013) but not in women (ß = 127.33, *p* = 0.865). Stopping volunteering remained not significant in men (ß = -751.31, *p* = 0.133) and women (ß = -475.53 *p* = 0.474).

In the adjusted asymmetric FE regression model including the total sample, we still found an indication towards an association between starting to volunteer and increased PA (*p* = 0.052) (Table 2). Stopping volunteering remained not significant (*p* = 0.543) among the total sample in the adjusted analyses. As also observed in the unadjusted FE regression models, the stratification by sex revealed a statistically significant increase in PA when starting to volunteer in men (*p* = 0.016) but not in women (*p* = 0.832). Stopping volunteering in the adjusted analyses remained not significant in both men (*p* = 0.583) and women (*p* = 0.845).

**Table 2:** Determinants of changes in physical activity. Results from adjusted fixed effects regression analyses

	All	Men	Women
**Start volunteering**	1,078.93 + (554.69)	1,751.54* (724.50)	187.25 (881.68)
**Stop volunteering**	-285.61 (469.88)	-320.76 (583.55)	-158.96 (813.88)
**Age**	-98.58 + (52.62)	-144.82* (69.07)	-32.12 (82.19)
**Marital status** (Ref. = married, living together)			
Married living apart	-1,039.39 (2,069.47)	-2,187.51 (2,445.91)	2,150.33 (2,149.35)
Divorced	1,716.36 (2,168.67)	745.69 (2,812.33)	3,047.54*** (375.10)
Widowed	-294.07 (737.95)	579.37 (839.91)	-1,232.77 (1,317.73)
Single	1,789.02*** (509.86)	1,731.16*** (320.71)	800.70 (1,800.62)
**OECD monthly equivalence income**	0.07 (0.26)	-0.06 (0.40)	0.22 (0.34)
**Self-efficacy from 1 (low) to 4 (high)**	652.05 + (373.56)	482.72 (512.59)	795.75 (558.87)
**Self-rated health** (Ref. = very good)			
Good	-147.92 (475.16)	134.34 (602.10)	-580.18 (755.03)
Average	-433.81 (548.77)	-50.46 (697.30)	-936.86 (885.64)
Bad	-680.87 (704.32)	-850.71 (866.80)	-550.04 (1,167.23)
Very bad	-1,174.35 (1,124.97)	-1,936.18 (1,610.87)	-192.76 (1,340.67)
**Sum of morbidities (from 0 to 11)**	-56.79 (104.21)	65.80 (115.74)	-234.51 (186.68)
**Physical functioning (SF36 subscale) from 0 (worst) to 100 (best)**	12.59 (8.51)	11.34 (10.49)	14.86 (14.25)
**Incidence of accident or disease** (Ref. = disease)			
Accident	864.81 (540.77)	1,106.20 (801.12)	734.16 (780.71)
Both, accident and disease	343.44 (641.12)	401.97 (850.01)	426.88 (944.51)
No, neither	91.51 (273.69)	134.59 (327.13)	25.81 (494.26)
**Constant**	10,228.43*	13,764.99*	5,687.53
	(4,279.45)	(5,688.68)	(6,650.21)
**Observations**	5,682	3,172	2,510
**Number of individuals**	4,075	2,242	1,833
**R**^**2**^	0.02	0.03	0.02

^***^ *p* < 0.001, ** *p* < 0.01, * *p* < 0.05, + *p* < 0.10

Unstandardized regression coefficients are displayed. Robust standard errors in parentheses

*Ref* Reference group

The observed differences in the estimates for a positive association between starting to volunteer and changes in PA in the stratified analyses by sex were not statistically significant (*p* = 0.233). This was investigated using an FE regression that included an interaction term between starting to volunteer and sex.

## Discussion

### Key results

Based on a large nationally representative sample, our aim was to clarify whether starting and stopping to volunteer is associated with changes in physical activity in older women and men. Our hypothesis that starting to volunteer is associated with an increase in PA, and stopping volunteering with a decrease in PA, was only partly confirmed in asymmetric FE regression analysis. The change in PA only reached statistically significance in men starting to volunteer. Men who started to volunteer increased their PA per week of eg. about 7.5 h of moderate PA.

### Interpretation

As in previous studies, we investigated the association between volunteering and PA [9, 10, 30]. However, to the best of our knowledge, this is the first study investigating the association between changes in volunteering status and changes in PA, with special focus on the different effect sizes for starting and stopping volunteering, in combination with a stratification for sex.

The three studies on the association between volunteering and PA included in a systematic review could not conclusively identify an association. One of the studies only included women [16]. Another investigated PA as mediator between the association between productive engagement, including volunteering, and no longer driving a car [31]. The third study focused on physical functioning rather than PA [32]. The large differences in the included study population, design, focus, and details may explain the inconclusive result of the systematic review. In future studies, more focus should be given to the details and the context.

In an RCT that investigated the association between volunteering and PA, predominantly African American older adults were included. The intervention group assisted children at school for at least 15 h per week [9]. Besides cultural dissimilarities in this context, the major difference between that RCT and our study lies in the comparison. While the RCT performed group comparison, we investigated within-individual changes. Nonetheless, the results point in the same direction, highlighting a positive association between volunteering and PA.

Another difference between the RCT and our study is the broader context of this work. While in the RCT, one specific task of volunteering was investigated, we included any officially volunteering task. However, we did not include details on the time spent in volunteering; but most volunteers spend less than 15 h, which was the minimum in the presented RCT [12]. These two factors: tasks associated with the volunteering, and the time spent volunteering, could be the reason for the insignificance of our observed findings. Future studies should therefore consider the task of, and time spent, volunteering when evaluating associations.

Furthermore, the underlying organisation and type of volunteering might be the reason for differences between men and women, who are likely to have different preferences in terms of volunteering [16]. While men are more likely to volunteer in sports and recreational organisations, women prefer to volunteer in religious organisations; volunteering for these different organisations include different levels of PA [16]. On the other hand, a lack of power may explain the lack of statistically significant differences between both sexes observed in our additional analysis that investigated the interaction between starting volunteering and sex. Another explanation for differences in our sex stratified analyses—but no statistically significant differences between sex—might be that in our sample, women were more active than men. This difference in levels of physical activity does not correspond with the existing literature [4]. Thus, we assume that women, independent of their volunteering status, already had a high physical activity level, and therefore increasing the amount of physical activity might be particularly challenging for these women.

The difference in effect size and significance, at least in men, between starting and stopping volunteering and its association with PA, might be explained by the fact that volunteers stop this role due to competing time demands. That is, they may replace their volunteering with another duty or task e.g. taking care of grandchildren, which may require the same amount of PA.

In summary, starting to volunteer was associated with an increase in PA in men, while stopping was not significantly associated with a decrease in PA. Thus, in addition to the benefits of volunteering for health status, as reported in previous studies, it also supports enhanced PA in men [9, 13].

### Strengths and limitations

To the best of our knowledge, this is the first study to investigate the association between starting and stopping volunteering and changes in PA. Our finding that starting to volunteer is associated with a larger increase in PA, than the decrease in PA when stopping volunteering, in men has never been reported before. Moreover, our data from a large national representative longitudinal study is a major strength of the study. It allowed us to investigate associations of rarely investigated phenomena, such as starting to volunteer in older adults.

The use of FE regressions, which use within-individual changes, and which eliminates the influence of time-constant (both, observed and unobserved) factors on the estimates, was an additional strength in this study.

A limitation of the study is the use of self-reported information, which is always prone to recall bias, and an overestimation of desired behaviour, such as PA. This effect, however, could be small, as it is likely that recall ability and overestimation of desired behaviour is constant within individuals over time, and thus would not have influenced the findings drawn from within-individual change observations.

Special attention should be paid to our variables of interest: volunteering and PA. Participants were coded as volunteering if they performed any task and any timespan of volunteering within an organisation. This means that there is limited information on the type of volunteering and time spent volunteering, leading to potentially significant heterogeneity in volunteers. However, the aim of this study was to investigate if volunteering in general leads to increases in physical activity. Nonetheless, we would recommend to include information on the task, time spent, and type of volunteering organisation in future studies. Another limitation regarding the change in volunteering status is that we do not know the exact time when the participant started or stopped volunteering during the study period and if this happened at the same time as the change in physical activity occurred. Measuring our dependent variable (PA) in a valid and reliable way is well-recognised as difficult [33]. However, a systematic review concluded that the IPAQ is suitable for evaluating within individual changes [34].

Finally, previous research has shown that a small selection bias exists in the DEAS study [17]. Despite the design of this representative national cohort study, the low response rate might have slightly skewed the inclusion of participants in the present evaluation, with a slightly higher likelihood of participation among men, participants living in rural regions, and among people aged between 40 and 54 years [17]. However, the DEAS study is disproportionally stratified by age, region and gender [17]. This is done to oversample groups expected to contribute to panel attrition, so as to ensure the sample to remain representative in the upcoming waves.

## Conclusion and future research

In conclusion, our results indicate an association between starting to volunteer and increased physical activity in older men. Therefore, clinicians, especially general practitioners, geriatrists and physiotherapists, may be encouraged to advise older men to start volunteering to enable and support active, healthy living and aging. The findings of our study hold true for the German context, as the sample is very similar to the population of Germany [17].

## Data Availability

The anonymised data used in this study were obtained from the “Deutsches Zentrum für Altersfragen”. Access can be obtained after application, please visit the following website for further information Access to DEAS data: Deutsches Zentrum für Altersfragen (dza.de).

## References

[1] Booth FW, Roberts CK, Laye MJ (2011). Lack of exercise is a major cause of chronic diseases. Compr Physiol.

[2] Pedersen BK, Saltin B (2015). Exercise as medicine–evidence for prescribing exercise as therapy in 26 different chronic diseases. Scand J Med Sci Sports.

[3] Hamer M, Lavoie KL, Bacon SL (2014). Taking up physical activity in later life and healthy ageing: the English longitudinal study of ageing. Br J Sports Med.

[4] Sun F, Norman IJ, While AE (2013). Physical activity in older people: a systematic review. BMC Public Health.

[5] French DP, Olander EK, Chisholm A, Mc SJ (2014). Which behaviour change techniques are most effective at increasing older adults’ self-efficacy and physical activity behaviour? A systematic review. Ann Behav Med.

[6] Greaves CJ, Sheppard KE, Abraham C, Hardeman W, Roden M, Evans PH (2011). Systematic review of reviews of intervention components associated with increased effectiveness in dietary and physical activity interventions. BMC Public Health.

[7] Choi J, Lee M, Lee J-k, Kang D, Choi J-Y. Correlates associated with participation in physical activity among adults: a systematic review of reviews and update. BMC Public Health. 2017;17(1):1–13. 10.1186/s12889-017-4255-2 PMC5404309 28438146

[8] Koeneman MA, Verheijden MW, Chinapaw MJ, Hopman-Rock M (2011). Determinants of physical activity and exercise in healthy older adults: a systematic review. Int J Behav Nutr Phys Act.

[9] Von Bonsdorff MB, Rantanen T (2011). Benefits of formal voluntary work among older people. A review. Aging Clin Exp Res.

[10] Niebuur J, van Lente L, Liefbroer AC, Steverink N, Smidt N (2018). Determinants of participation in voluntary work: A systematic review and meta-analysis of longitudinal cohort studies. BMC Public Health.

[11] Zahl der freiwillig Engagierten in Deutschland weiterhin hoch: Bundesministerium für Familie, Senioren, Frauen und Jugend. [Available from: https://www.bmfsfj.de/bmfsfj/aktuelles/alle-meldungen/zahl-der-freiwillig-engagierten-in-deutschland-weiterhin-hoch-176842.

[12] von Rosenbladt B. Freiwilliges Engagement in Deutschland. Freiwilligensurvey 1999: Ergebnisse der Repräsentativerhebung zu Ehrenamt, Freiwilligenarbeit und bürgerschaftlichem Engagement Band 1: Gesamtbericht: Springer-Verlag; 2009.

[13] Russell AR, Nyame-Mensah A, de Wit A, Handy F (2019). Volunteering and wellbeing among ageing adults: A longitudinal analysis. VOLUNTAS Int. J. Nonprofit Volunt.

[14] Tan EJ, Xue Q-L, Li T, Carlson MC, Fried LP (2006). Volunteering: a physical activity intervention for older adults—the experience Corps® program in Baltimore. J Urban Health.

[15] Onyx J, Warburton J (2003). Volunteering and health among older people: A review. Australas J Ageing.

[16] Parkinson L, Warburton J, Sibbritt D, Byles J (2010). Volunteering and older women: Psychosocial and health predictors of participation. Aging Ment Health.

[17] Klaus D, Engstler H, Mahne K, Wolff JK, Simonson J, Wurm S (2017). Cohort profile the German ageing survey (DEAS). Int. J. Epidemiol..

[18] Mahne K, Katharina Wolff J, Simonson J, Tesch-Römer C. Altern im Wandel: Zwei Jahrzehnte Deutscher Alterssurvey (DEAS): Springer Nature; 2017.

[19] Schiel S, Dickmann C, Aust F. Methodenbericht Deutscher Alterssurvey (DEAS): 4. Befragungswelle Panelbefragung. 2011;2011.

[20] Hsu CH, Tung H-H, Clinciu DL, Chen L-K, Yin W-H, Iqbal U (2017). Physical activity: A primary health quality determinant among community-dwelling geriatric women in Taiwan. Int J Qual Health Care.

[21] Mazya AL, Garvin P, Ekdahl AW (2019). Outpatient comprehensive geriatric assessment: effects on frailty and mortality in old people with multimorbidity and high health care utilization. Aging Clin Exp Res.

[22] Hagströmer M, Oja P, Sjöström M (2006). The International Physical Activity Questionnaire (IPAQ): a study of concurrent and construct validity. Public Health Nutr.

[23] Booth ML, Owen N, Bauman AE, Gore CJ (1996). Retest reliability of recall measures of leisure-time physical activity in Australian adults. Int J Epidemiol.

[24] International Physical Activity Questionnaire.[Available from: https://sites.google.com/site/theipaq/home.

[25] Flennert M, König H-H, Hajek A (2019). The association between voluntary work and health care use among older adults in Germany. BMC Health Serv Res.

[26] Stuck AE, Walthert JM, Nikolaus T, Büla CJ, Hohmann C, Beck JC (1999). Risk factors for functional status decline in community-living elderly people: a systematic literature review. Soc Sci Med.

[27] Ware Jr JE, Sherbourne CD. The MOS 36-item short-form health survey (SF-36) I Conceptual framework and item selection. Med Care. 1992:473–83 1593914

[28] Cameron AC, Trivedi PK. Microeconometrics: methods and applications: Cambridge university press; 2005. 699 p.

[29] Allison PD (2019). Asymmetric fixed-effects models for panel data. Socius.

[30] Librett J, Yore MM, Buchner DM, Schmid TL. Take pride in America’s health: volunteering as a gateway to physical activity. Am J Health Educ. 2005;36(1):8–13.

[31] Curl AL, Stowe JD, Cooney TM, Proulx CM (2014). Giving up the keys: How driving cessation affects engagement in later life. Gerontologist.

[32] Cramm JM, Nieboer AP (2015). Background characteristics, resources and volunteering among older adults (aged≥ 70 years) in the community: A longitudinal study. Geriatr Gerontol Int.

[33] Sylvia LG, Bernstein EE, Hubbard JL, Keating L, Anderson EJ (2014). Practical guide to measuring physical activity. J Acad Nutr Diet.

[34] Lee PH, Macfarlane DJ, Lam TH, Stewart SM (2011). Validity of the international physical activity questionnaire short form (IPAQ-SF): A systematic review. Int J Behav Nutr Phys Act.

